# Mode of interaction of the Gαo subunit of heterotrimeric G proteins with the GoLoco1 motif of *Drosophila* Pins is determined by guanine nucleotides

**DOI:** 10.1042/BSR20150201

**Published:** 2015-11-26

**Authors:** Anne-Marie Lüchtenborg, Vladimir Purvanov, Bogdan S. Melnik, Simon Becker, Vladimir L. Katanaev

**Affiliations:** * Department of Pharmacology and Toxicology, University of Lausanne, CH-1011, Switzerland; † Biotechnology Institute Thurgau, Kreuzlingen, CH-8280, Switzerland; ‡ Institute of Protein Research, Russian Academy of Sciences, 142290 Pushchino, Russian Federation; § Department of Biology, University of Konstanz, 78457 Konstanz, Germany; ║ School of Biomedicine, Far Eastern Federal University, Vladivostok 690922, Russian Federation

**Keywords:** asymmetric cell division, FRET, GoLoco, heterotrimeric G proteins, nucleotide exchange

## Abstract

*Drosophila* GoLoco motif-containing protein Pins is unusual in its highly efficient interaction with both GDP- and the GTP-loaded forms of the α-subunit of the heterotrimeric Go protein. We analysed the interactions of Gαo in its two nucleotide forms with GoLoco1–the first of the three GoLoco domains of Pins–and the possible structures of the resulting complexes, through combination of conventional fluorescence and FRET measurements as well as through molecular modelling. Our data suggest that the orientation of the GoLoco1 motif on Gαo significantly differs between the two nucleotide states of the latter. In other words, a rotation of the GoLoco1 peptide in respect with Gαo must accompany the nucleotide exchange in Gαo. The sterical hindrance requiring such a rotation probably contributes to the guanine nucleotide exchange inhibitor activity of GoLoco1 and Pins as a whole. Our data have important implications for the mechanisms of Pins regulation in the process of asymmetric cell divisions.

## Abstract (precis)

*Drosophila* GoLoco-containing protein Pins atypically interacts both with GDP- and GTP-loaded forms of Gαo. We show that Pins' GoLoco1 domain ‘rotates’ on Gαo depending on its nucleotide state. This mechanism has important implications for asymmetric cell divisions.

## INTRODUCTION

Heterotrimeric G proteins mediate signalling by G protein-coupled receptors (GPCRs), the biggest receptor family in the animal kingdom [1]. The α-subunits of heterotrimeric G proteins determine the specificity in GPCR signal transduction and interact with various effectors [2]. GoLoco motifs present in many animal proteins specifically bind to α-subunits of the Gi/o subclass of heterotrimeric G proteins [3]. We have demonstrated that in *Drosophila*, the Go protein links the polarizing information provided by the GPCR Frizzled with the GoLoco-containing protein Pins to regulate the process of asymmetric cell divisions in the sensory organ lineage [4,5]. Atypically for GoLoco–Gα interactions, Pins efficiently interacts with both the GDP-loaded (inactive) and the GTP-loaded (activated) forms of Gαo [4–6]; in other proteins, the exclusive interaction of Gα-GDP with GoLoco motifs has been described [3]. Our findings have identified Pins as a target of the Gαo-mediated GPCR signalling [5], as opposed to other GoLoco motif-containing proteins which are believed to act as modulators of the Gα-subunits [3].

We have narrowed down the Gαo-GTP-interacting region of Pins to the GoLoco1 motif (the first of three GoLoco motifs present in Pins) [5]. Through a combination of biophysical approaches, we now characterize the interactions between GoLoco1 and the two nucleotide forms of Gαo. Our data shed light on the possible mechanisms of formation of these unusual complexes and suggest that the orientation of GoLoco1 on Gαo is determined by the nucleotide state of the latter. These findings have important implications for the molecular mechanisms regulating activity of the multi-domain Pins protein in the process of cell polarization downstream from GPCR-Gαo signalling.

## MATERIALS AND METHODS

### Site-specific mutagenesis

Site-specific mutagenesis of *Drosophila* Gαo (class II isoform) was performed through high-fidelity amplification (with Pfu DNA-polymerase, Fermentas) of the pQE32-Gαo plasmid [5] with the following oligonucleotides introducing the point mutations: for W132F, forward 5'-ccatgaaacgcctct'tc'caggccagg-agtgc-3', reverse 5'-cctggcctg'ga'agaggcgtttcatggcggcc-3'; for W212F, forward 5'-cgtaagaaat'tc'atacactgcttcgaagatg-3', reverse 5'-cagtgtat'ga'atttcttacgttccgagcgc-3'; for W259F, forward 5'-gtaacaacaaat'tc'ttcacggacacctcg-3', reverse 5'-ccgt-gaa'ga'atttgttgttacagatcgag-3'. PCR products were treated with DpnI to remove the methylated template, purified by a gel-extraction kit (Peqlab) and used for bacterial transformation. The resulting plasmids were sequence-verified.

### Preparation of Gαo proteins and GoLoco peptides

Hexahistidine-tagged *Drosophila* Gαo, Gαo[W132F; W212F] and Gαo[W132F; W259F] were purified following [5]. In brief, Top10 bacteria transformed with the corresponding construct were grown in LB media until an OD_600_=0.6, expression was induced with 0.1 mM IPTG and cells further grown overnight at room temperature. Cells were harvested, resuspended in PBS supplemented with Complete Protease Inhibitor cocktail (Roche) and lysed with Lysozyme and sonication. Binding of the His_6_ tagged proteins to the Ni-NTA agarose was conducted in 10 mM Imidazole in PBS. After washing in 20 mM Imidazole/PBS the proteins were eluted in 200–250 mM Imidazole/PBS. Their specific activities varied from 20 to 60%, and were determined as described [5]. Preloading of purified Gαo with GDP or GTPγS was performed for 30 min as described [5]; from parallel BODIPY-GTPγS binding experiments the loading efficiency of Gαo, measured in percentage of *B*_max_, was determined as >97%. The 36-mer GoLoco1 peptide and the GoLoco1 peptide with the dansyl (5-(dimethylamino)naphthalene-1-sulfonyl) group attached to the side chain of the N-terminal lysine were synthesized by Pepscan Presto BV. Non-dansylated GoLoco1 was dissolved in water, dansyl-GoLoco1 was dissolved at 10 mM in 50% propan-2-ol and stored at -20°C.

### Fluorescence measurements

Fluorescence measurements were performed at RT using the Biotek SynergyMx plate reader. For all experiments, the Gαo subunits were pre-incubated with 1 mM GDP or GTPγS for >30 min at RT. For FRET measurements, preloaded Gαo was added in a final concentration of 10 μM to increasing concentration of dansylated GoLoco1 in HKB (50 mM Hepes-KOH, 100 mM KCl, 10 mM NaCl, 2 mM EGTA, 5 mM MgCl_2_, 1 mM DTT) and incubated for 30 min before measurement. Trp-to-dansyl FRET was measured at the wavelength of 535 nm after excitation at 280 nm. Similarly, for the tryptophan fluorescence measurement, 5 μM preloaded Gαo were incubated for 30 min with unlabelled or dansylated GoLoco1. Tryptophan fluorescence was measured at 330 nm after excitation at 280 nm. For the competition experiment, preloaded Gαo was added at the 1:1 ratio (final concentration of 5–10 μM) to dansylated GoLoco1 and pre-incubated for 10 min. Subsequently, increasing concentrations of the unlabelled GoLoco1 peptide in HKB were added and incubated for 30 min to allow establishment of the equilibrium. Trp-to-dansyl FRET was measured as above.

*K*_d_ and *K*_i_ values were calculated using the Prism program. To calculate the *E* values (FRET efficiency), background fluorescence in the absence of added Gαo was subtracted from the absolute data of tryptophan fluorescence and *E* calculated as (1 - *F*_D-A_/*F*_D_), where *F*_D-A_ was the measured fluorescence of 5 μM Gαo–dansyl-GoLoco1 complex, and *F*_D_ that of 5 μM Gαo–GoLoco1 complex. The distance *r* between the donor and acceptor fluorophores was calculated using the Förster equation 1/*E*=1 + (*r*/*R*_0_)^6^. *R*_0_ depends on the orientation factor *κ*^2^ of the dipole–dipole interaction which is a function of the specific dipole orientation of donor and acceptor. In case of dynamic isotropic movement of the labels, *κ*^2^ is 2/3, which is the case for tryptophan and dansyl that are covalently linked with a single atom bond [7]. With this assumption, *R*_0_ for the tryptophan/dansyl pair is 21 Å (1 Å=0.1 nm) [8].

### Structure modelling

Structure modelling was performed using the PDB entry 1KJY describing the GDP form of human Gαi in complex with the GoLoco motif of RGS14 [9,10]. As human Gαi and *Drosophila* Gαo sequences share 71% identical residues, modelling of Gαo could be performed using SwissModel [11]. The Coot package [12] was used for introducing the sequence of the Pins GoLoco motif, and for changing GDP into GTP, guided by the GDP:AlF_4_^+^ coordinates of the mouse Gαo [13]. To optimize the resulting interactions, energy minimization calculations were performed using CNS 1.2 [14] on a Linux workstation using a force field with explicit hydrogens, corresponding to the files protein-allhdg.top and protein-allhdg.param. The images (Figures 3A and 3B) were produced using Maestro (Version 9.0.211, Schrödinger Inc).

## RESULTS AND DISCUSSION

Previously we have demonstrated that the GoLoco1 domain of *Drosophila* Pins efficiently interacts with both the GDP-loaded and the GTP-loaded form of Gαo [5]. In order to investigate the unusual interaction of the GoLoco1 domain with Gαo-GTP in detail, we performed a series of FRET experiments. To this end, we synthetized the extended 35 amino acid long Pins GoLoco1 peptide and its fluorescent analogue, where the side chain of the N-terminal lysine was dansylated (*d*GoLoco1, Figure 1A). The dansyl group added on proteins/peptides has been extensively used as an acceptor of energy from excited tryptophans in FRET experiments [15,16]. We first confirmed the binding of GoLoco1 with Gαo, taking advantage of the fact that FRET occurs between the donor tryptophan and the acceptor dansyl in case donor and acceptor are in close proximity. Upon excitement of tryptophan, a FRET signal should therefore only been seen upon binding of *d*GoLoco1 to Gαo, and the strength of the signal should be proportional to the distance between the tryptophan donor and the dansyl acceptor. Indeed, we detect a robust FRET signal to *d*GoLoco1 from both nucleotide states of Gαo, although the signal from Gαo-GTPγS was several folds lower than from Gαo-GDP (Figure 1B). However, analysis of the saturation curves shows that the affinity of *d*GoLoco1 to the two nucleotide forms of Gαo is similar (Figure 1C): *K*_d_=4.6 μM for Gαo-GDP–*d*GoLoco1 and *K*_d_=2.1 μM for Gαo-GTPγS–*d*GoLoco1. Further, *d*GoLoco1 from both complexes can be similarly outcompeted by the non-dansylated GoLoco1 peptide (Figure 1D). The *K*_i_ values resulting from these competition experiments are 17.6 μM for Gαo-GDP and 10.6 μM for Gαo-GTPγS. The finding that *K*_i_ for unlabelled GoLoco1 is somewhat higher than *K*_d_ for *d*GoLoco1 may indicate that the hydrophobic dansyl group increases the affinity of the GoLoco1 peptide to Gαo. In any regard, we conclude that the binding affinity of the GoLoco1 peptide of Pins to Gαo is similar for the two nucleotide states of the G protein, but that the proximity of the N-terminus of GoLoco1 to Gαo differs in the two nucleotide states.

**Figure 1:**
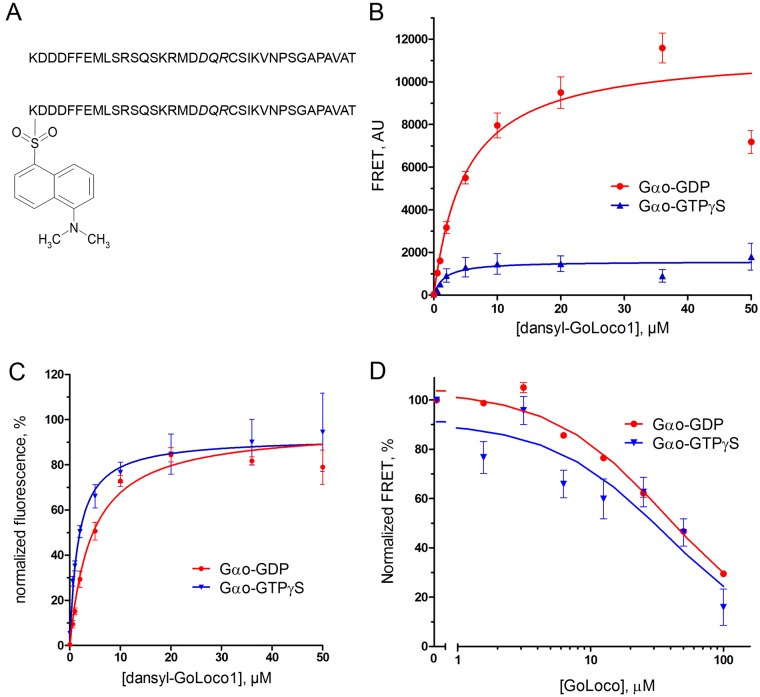
GoLoco1 binds with similar affinity but different orientation to Gαo-GTP and Gαo-GDP (**A**) Sequence of the GoLoco1 peptide of Pins and schematic representation of its dansylated variant, with the dansyl group attached to the side chain of Lys^1^ of this sequence. The DQR triad conserved among GoLoco domains and mediating the interaction with the guanine nucleotide within Gα is shown in italics. (**B** and **C**) Saturation curves of binding of increasing concentrations of dansyl-GoLoco1 to Gαo preloaded with GDP or GTPγS as measured by FRET of tryptophan to dansyl indicate comparable binding of Gαo-GDP and Gαo-GTPγS to the peptide. The data is presented as arbitrary fluorescence units (**B**) and normalized fluorescence units (**C**). (**D**) Competition experiment using increasing concentrations of the unlabelled GoLoco1 peptide decreasing the normalized FRET signal from Gαo-GDP and Gαo-GTPγS complexes with the dansylated peptide. All the data are presented as mean ± S.E.M., *n*=7.

The Förster critical transfer distance *R*_0_ for the tryptophan/dansyl pair, the distance at which 50% quenching of the tryptophan fluorescence is induced due to the proximity of the dansyl acceptor, is 21 Å assuming a value of 2/3 for the orientation factor *κ*^2^ [8]. By the decrease in tryptophan fluorescence due to energy transfer to the acceptor dansyl in proximity, the distance between the donor and acceptor molecules can be calculated using the Förster equation 1/*E*=1 + (*r*/*R*_0_)^6^, where *E* is the FRET efficiency and *r* is the distance between donor and acceptor (see Materials and Methods section) [15,17].

No tryptophan residues are present in the GoLoco1 peptide (Figure 1A). *Drosophila* Gαo contains three tryptophan residues: Trp^132^ located in the helical domain far from the GoLoco1-interacting region, Trp^212^ in the switch II region and Trp^259^ in the α3-helix. The latter two regions of Gαo mediate the binding of GoLoco1 (see later), and thus their tryptophan residues might be involved in the FRET. To investigate this issue in more detail, we substituted tryptophan residues with phenylalanines, producing two double mutant Gαo versions with Trp^212^ or Trp^259^ as the only remaining tryptophan. Such mutations on Trp^132^ and Trp^212^ have been performed previously on other Gα-subunits and shown not to affect the overall activity of the G proteins [18,19]. The W259F substitution was also expected to produce no significant functional differences, as this substitution naturally occurs in the class I isoform of mammalian Gαo.

We next measured tryptophan fluorescence of wild-type and the two double mutant forms of Gαo, Gαo[W132F; W212F] and Gαo[W132F; W259F], preloaded with GDP or GTPγS, in complexes with the unlabelled GoLoco1 and *d*GoLoco1 peptides.

We see a robust decrease in tryptophan fluorescence of all three Gαo-GDP forms in the presence of *d*GoLoco1 but not unlabelled GoLoco1 (Figure 2). In contrast, no or low decrease can be detected for the Gαo-GTPγS–*d*GoLoco1 complexes (Figure 2), confirming the presented above (see Figure 1B) and more sensitive FRET measurements which suggest the large distance between tryptophan residues and dansyl for the GTP-loaded forms. A detectable decrease can only be seen for the GTP-loaded form with Trp^212^ as the only remaining tryptophan (Figure 2B), suggesting the closest proximity of this residue of Gαo-GTPγS to the dansyl. We think that no decrease in tryptophan fluorescence could be detected for the Gαo-GTPγS–*d*GoLoco1 complexes using wild-type Gαo due to the masking of the effect of Trp^212^ by the other two, non-participating tryptophan residues.

**Figure 2:**
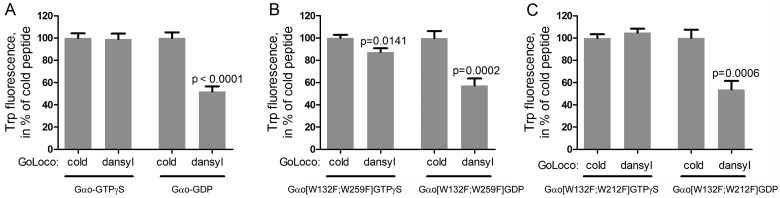
The N-terminal lysine is more distant when binding to Gαo-GTP (**A**) Tryptophan fluorescence of wild-type Gαo preloaded with either GDP or GTPγS in presence of the unlabelled or dansylated GoLoco1 peptide. (**B**) Tryptophan fluorescence of the mutant Gαo[W132F;W259F] preloaded with the guanine nucleotides as indicated. (**C**) Tryptophan fluorescence of the mutant Gαo[W132F;W212F] preloaded with the guanine nucleotides as indicated. Data are normalized to the fluorescence in presence of the unlabelled GoLoco1 peptide and present as mean ± S.E.M., *n*=5. *P*-values calculated by the Student *t*-test are given for the statistically significant differences.

The FRET efficiency for the Trp^212^-only mutant form (=Gαo[W132F;W259F] mutant form) is calculated as *E*_GTPγS_=0.13 for Gαo-GTPγS, and as *E*_GDP_=0.54 for Gαo-GDP. Using the Förster equation, we then calculate the distance between Trp^212^ of Gαo-GTPγS and the dansyl group of GoLoco1 as *r*_GTPγS_ ∼ 30 Å. Similar calculation for Gαo-GDP yields *r*_GDP_ *∼* 21 Å.

Similar analysis of the data for the Trp^259^-only mutant (=Gαo[W132F;W212F] mutant form) (Figure 3C) produce for Gαo-GDP *E*_GDP_=0.48 and *r*_GDP_ *∼* 21 Å. In case of Gαo-GTPγS, no reduction in tryptophan fluorescence is observed. Given the relationship of *R*_0_ and the distance of acceptor and donor *r* with the power of 6, one can assume a separation of tryptophan and dansyl of >31.5 Å [7]. These data are summarized in Table 1.

**Figure 3:**
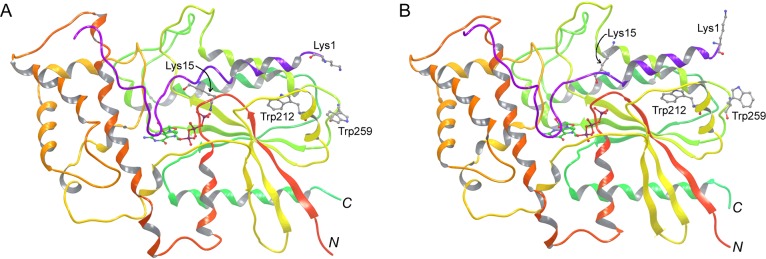
Molecular modelling and analysis of the Gαo–GoLoco1 complex (**A**) Model of the Gαo-GTP–GoLoco1 complex built using the 1KJY structure of the homologous mammalian complex of Gαi1-GDP with the GoLoco region of RGS14 as the basis. (**B**) The model built using the 2OM2 structure of the same complex. In both models GoLoco1 is shown in blue-magenta, N-terminus to the right. The helical domain of Gαo is left, the catalytic domain is right, with the GTP placed in the centre of the cleft between the two domains. Side chains of four amino acids are shown: Lys^1^ and Lys^15^ of GoLoco1, Trp^212^ of switch II of Gαo and Trp^259^ of α3-helix of Gαo. Lys^15^ is highlighted to illustrate that the whole α-helix of GoLoco1 (and not just Lys^1^) rotates from one conformation to the other.

**Table 1:** FRET efficiency and distance Data are given as mean ± S.E.M., *n*=5. N.P.–not possible to calculate.

G-protein	*E*	*R*
Gαo		
GTPγS-bound	−0.022±0.072	N.P.
GDP-bound	0.474±0.028	N.P.
GαoW132F-W212F		
GTPγS-bound	−0.017±0.048	>31.5 Å
GDP-bound	0.478±0.054	21.38±0.77 Å
GαoW132F-W259F		
GTPγS-bound	0.127±0.029	29.66±1.41 Å
GDP-bound	0.544±0.100	20.64±1.64 Å

Thus, we conclude that, for Gαo-GDP, the distance between Trp^212^ and the N-terminus of GoLoco1 is *∼*21 Å and equals the distance from Trp^259^ and the N-terminus of GoLoco1. In contrast, for the GTP-loaded form of Gαo, this distance for Trp^212^ is *∼*30 Å, and for Trp^259^ it is larger than 31.5 Å. These findings argue for a substantially distinct conformation of the Gαo–GoLoco1 complexes depending on the nucleotide state of the G protein.

To visualize the possible differences in the conformation of the Gαo–GoLoco1 complexes in the presence of GDP compared with GTP, we performed molecular modelling of the complexes. The structure of a homologous human complex of Gαi1-GDP with the GoLoco region of RGS14 [9] was used as the basis for our model of the *Drosophila* Gαo-GTP–GoLoco1 complex; the model was further optimized using the structure of mouse Gαo-GDP:AlF_4_^+^ [13]. The resulting structure of the Gαo-GTP–GoLoco1 interaction shows the side chain of N-terminal lysine of GoLoco1 in close proximity to Trp^212^ and Trp^259^ of Gαo (Figure 3A), with the distances from the side chain of GoLoco's Lys^1^ to Gαo' Trp^212^ and Trp^259^ being 4.5 and 9.2 Å, respectively. However, when a different structure of the same Gαi1–GoLoco complex [10] was used as the basis for modelling, the side chain of Lys^1^ was found to point away from the protein (Figure 3B), resulting in the distances to Trp^212^ and Trp^259^ being 11.7 and 13.6 Å, respectively. In fact, quite significant differences in the orientation of the first α-helix of RGS14' GoLoco could be seen between the two structures [9,10] explaining the different rotation of the modelled α-helix of Pins' GoLoco1 motif (Figures 3A and 3B).

We do not know the reason for the difference between the two Gαi1–GoLoco structures [9,10]. However, we note that the two predicted rotations of the *Drosophila* Pins' GoLoco1 in the complex with Gαo (Figures 3A and 3B) reflect the two possible conformations of the complex which we predicted from our FRET experiments. Taking into consideration the dimension of the dansyl group which is *∼*4.7 Å, we suggest that the model of Figure 3(A) being close to the GDP data, and Figure 3(B) being close to the GTPγS data (Table 1).

These two conformations of GoLoco1 require quite a substantial rotation of the α-helix (Figures 3A and 3B). The existence of such a rotation currently remains a possibility, which must be verified by direct structural analysis. In any case, our data demonstrate that the conformation of the GoLoco1 region of Pins on Gαo is substantially different depending on the nucleotide state of Gαo. These findings have quite important implications for the biochemistry and physiology of the GPCR-Gα-Pins signalling. Indeed, Pins and other GoLoco domain-containing proteins possess an activity inhibiting the nucleotide exchange on Gα-subunits [3,5]. Stabilization of the β-phosphate of GDP by a conserved arginine within the DQR triad (see Figure 1A) of the GoLoco sequence has been proposed as the mechanism of this inhibition [9]. We here wish to propose the sterical hindrance, required to perform the rotation (or another structural reorganization) of the GoLoco peptide on Gα for the nucleotide exchange, as an additional leverage to this inhibition, relevant at least for the *Drosophila* Gαo–Pins interaction.

Further, the different rotation/organization of the GoLoco1 peptide in respect with Gαo, observed in the two nucleotide states of the G protein, is likely to have significant consequences to the signal transduction mediated by the Gαo-Pins interaction. Indeed, the multidomain Pins binds a number of other proteins, including the microtubule anchoring protein NuMA [20]. Coordinated binding of Pins to NuMA and Gα has been reported [21,22]. We can predict that the ability of Pins to coordinate asymmetric cell divisions is regulated differently by the GDP- and GTP-bound forms of Gαo. This is likely to provide important regulatory mechanisms for different GPCR-Gαo-Pins signalling schemes, such as Frizzled-Gαo-Pins cascade in case of asymmetric cell divisions in the sensory organ lineage [5] and in Tre1-Gαo-Pins cascade in asymmetric neuroblast divisions [6].

## Abbreviations

GPCR: G protein-coupled receptor
